# Ionomic and Metabolomic Analyses Reveal Different Response Mechanisms to Saline–Alkali Stress Between *Suaeda salsa* Community and *Puccinellia tenuiflora* Community

**DOI:** 10.3389/fpls.2021.774284

**Published:** 2021-11-30

**Authors:** Qi Chen, Yan Jin, Zhonghua Zhang, Meng Cao, Guanyun Wei, Xiaorui Guo, Jian Zhang, Xueyan Lu, Zhonghua Tang

**Affiliations:** ^1^School of Life Sciences, Nantong University, Nantong, China; ^2^Key Laboratory of Plant Ecology, Northeast Forestry University, Harbin, China; ^3^Heilongjiang Institute of Green Food Science, Northeast Agricultural University, Harbin, China

**Keywords:** saline-alkali stress, *Suaeda salsa* community, *Puccinellia tenuiflora* community, ionomic, metabolomic

## Abstract

Soil salinization imposes severe stress to plants, inhibits plant growth, and severely limits agricultural productivity and land utilization. The response of a single plant to saline-alkali stress has been well investigated. However, the plant community that usually works as a group to defend against saline–alkali stress was neglected. To determine the functions of plant community, in our current work, *Suaeda salsa* (*S. salsa*) community and *Puccinellia tenuiflora* (*P. tenuiflora*) community, two communities that are widely distributed in Hulun Buir Grassland in Northeastern China, were selected as research objects. Ionomic and metabolomic were applied to compare the differences between *S. salsa* community and *P. tenuiflora* community from the aspects of ion transport and phenolic compound accumulation, respectively. Ionomic studies demonstrated that many macroelements, including potassium (K) and calcium (Ca), were highly accumulated in *S. salsa* community whereas microelement manganese (Mn) was highly accumulated in *P. tenuiflora* community. In *S. salsa* community, transportation of K to aboveground parts of plants helps to maintain high K^+^ and low Na^+^ concentrations whereas the accumulation of Ca triggers the salt overly sensitive (SOS)-Na^+^ system to efflux Na^+^. In *P. tenuiflora* community, enrichment of Mn in roots elevates the level of Mn-superoxide dismutase (SOD) and increases the resistance to saline–alkali stress. Metabolomic studies revealed the high levels of C6C1-compounds and C6C3C6-compounds in *S. salsa* community and also the high levels of C6C3-compounds in *P. tenuiflora* community. C6C1-compounds function as signaling molecules to defend against stress and may stimulate the accumulation of C6C3C6-compounds. C6C3-compounds contribute to the elimination of free radicals and the maintenance of cell morphology. Collectively, our findings determine the abundance of phenolic compounds and various elements in *S. salsa* community and *P. tenuiflora* community in Hulun Buir Grassland and we explored different responses of *S. salsa* community and *P. tenuiflora* community to cope with saline–alkali stress. Understanding of plant response strategies from the perspective of community teamwork may provide a feasible and novel way to transform salinization land.

## Introduction

According to the statistics of the United Nations Educational, Scientific and Cultural Organization (UNESCO) and the Food and Agriculture Organization of the United Nations (FAO), there are over 900 million ha of land with varying degrees of salinization worldwide. These salinized lands span more than 100 countries and account for 6.5% of the world’s total land area as well as 60% of the world’s total arable lands (Xia et al., 2013; Xu et al., 2020). More seriously, the area of salinized land is estimated to expand at a rate of 1.5 million ha per year. It is predicted that up to 50% of the land will be lost by 2050 (Xia et al., 2013; Xu et al., 2020).

Hulun Buir Grassland located in Northeast China is severely threatened by salinization due to man-made destruction and climate impact. In Hulun Buir Grassland, soil pH is higher than 9.8 and is classified as “saline-alkali” soil (Wang et al., 2007; Kobayashi et al., 2012). Saline–alkali soil normally means NaCl, Na_2_CO_3_, NaHCO_3_, Na_2_SO_4_, and NaOH (Yin et al., 2019). Saline–alkali stress leads to more serious damage than salt or alkali alone (Yu et al., 2013), impairs the physiological functions of plants, disturbs metabolic pathways, and adversely affects the growth of plants (Jia et al., 2019; Ye et al., 2019). The tolerance or defense responses against saline–alkali stress are composed of many cascades, including signal transduction, ion and osmotic homeostasis, antioxidant system, primary and secondary metabolisms, and transcription regulation. A large number of saline–alkali tolerant plants, such as *S. salsa*, *P. tenuiflora*, *Mesembryanthemum crystallinum*, and halophytes such as *Tamarix hispida*, *Leymus chinensis*, *Populus nigra*, *Limonium bicolor*, and *Puccinellia distans* have been investigated to explore the underlying tolerance or defense mechanisms (Kore-Eda et al., 2004; Taji et al., 2004; Wang et al., 2004; Ban, 2007; Wang et al., 2014; Zhao et al., 2016; Yin et al., 2019).

The majority of these researches focus on a single plant. However, in the natural ecological environment, plants resist saline–alkali stress via forming plant communities. *S. salsa* community and *P. tenuiflora* community were the dominant community types in Hulun Buir Grassland. *S. salsa* community is composed of its main constituent *S*. *salsa*, and also *Atriplex patens* and *Polygonum sibiricum*. *P. tenuiflora* community is composed of its main constituent *P*. *tenuiflor* and also *Plantago maritima* and *Carex reptabunda*. Investigating a single plant may result in loss of critical information, and that plant community functions as an entire group was particularly important. However, the systematic roles of plant community under saline–alkali stress have not been clearly reported.

Currently, ionomic and metabolomic-based technologies have been broadly employed to clarify the biological responses of various plants to saline–alkali stress, such as *Hordeum vulgare*, *Oryza sativa*, *Suaeda corniculata*, *Glycine max*, and *Zea mays* (Heleno et al., 2015; Pang et al., 2016; Guo et al., 2017; Yang et al., 2017). The application of ionomic and metabolomic offers a rational way to reveal changes under saline–alkali stress. It accelerates the understanding of the response mechanisms of plant salinization tolerance.

To overcome these limitations, instead of investigating a single plant, *S. salsa* community and *P. tenuiflora* community, two essential communities in Hulun Buir Grassland, were investigated here. Ionomic and metabolomic were used to compare the differences between *S. salsa* community and *P. tenuiflora* community. By studying these plant communities in groups, we attempt to build a response model to saline–alkali stress and decipher the resistance strategies of plants to saline–alkali stress from the perspective of community teamwork.

## Materials and Methods

### Plants and Growth Conditions

*Suaeda salsa* community and *P. tenuiflora* community in Hulun Buir grassland (115°31′00″–121°34′30″, 47°20′00″–50°50′30″) were collected as research materials from three plots. *S*. *salsa*, *A. patens*, and *P. sibiricum* were collected from *S. salsa* community. *P*. *tenuiflor*, *P. maritima*, and *C. reptabunda* were collected from *P. tenuiflora* community. Plants were divided into leaf, stem, and root, and then frozen in liquid nitrogen.

Soils were collected along the vertical length of 20 cm in depth and sieved through a 2-mm nylon sieve. Then 0.2 g dry sample of soil was treated with HF–HClO_4_–HNO_3_ method (Hseu et al., 2002; Bielicka-Daszkiewicz et al., 2012). The pH of the soil was measured with a glass electrode pH meter (pHM-2000, Rikakikai Co., Japan) in the saturation phase. The contents of carbonates and bicarbonates were measured by titration with HCl. The contents of chlorides were measured by titration with silver nitrate. The amounts of sulfates were measured by colorimetry (UV-140-02, Shimadzu, Japan) (Lu et al., 2021).

### Determination of Element Accumulation in Plants

Harvested plants were separated into leaves, stems, and roots and dried for 48 h at 60°C. Plant tissues were digested in concentrated HNO_3_ (95%) using a graphite plate (EH45A plus) at 130°C. The contents of potassium (K), calcium (Ca), sodium (Na), magnesium (Mg), manganese (Mn), copper (Cu), zinc (Zn), boron (B), nickel (Ni), and molybdenum (Mo) were determined by atomic absorption. Bioaccumulation factors (BFs) and transfer factors (TF) were calculated based on following formulas (Chen et al., 2018):


BFs = Mplant/Mmedium

TF = Mleaf/Mroot

*M*plant represents mass of detected elements in plant, *M*medium represents mass of detected elements in medium, *M*leaf represents mass of detected elements in leaves, and *M*root represents mass of detected elements in roots.

### Determination of Primary Metabolisms

Samples weighting 60 mg were extracted as previously described (Chen et al., 2017). After extraction, 1 μL prepared sample solution was injected into the Agilent 7890A-5975C gas chromatography-mass spectrometry (GC-MS) system (Agilent Corporation, United States). GC-MS analysis was carried out on a non-polar DB-5 capillary column (30 m × 250 μm I.D., J&W Scientific, Folsom, CA). High purity of helium was used as the carrier gas at a constant flow rate of 1.0 mL/min. The temperatures of injection and ion source were set to 305 and 230°C, respectively. Electron impact ionization (-70 eV) at full scan mode (m/z 30-600) was used, with an acquisition rate of 20 spectrum/second in the MS setting. QC sample was prepared by mixing aliquots of tissues samples to be a pooled sample.

Acquired data were analyzed by ChromaTOF software (v 4.34, LECO, St Joseph, MI). Internal standards and any known pseudo positive peaks were removed from the obtained data set. Data set was normalized using sum intensity of peaks in each sample. Obtained three-dimensional data sets included sample information, retention time, and peak intensities.

### Determination of Phenolic Compounds

The detection of phenolic compounds was conducted as previously described (Chen et al., 2021). Briefly, 1.0 g of liquid nitrogen pulverized sample was weighed and dissolved in 20 mL of methanol for extraction. Analysis was performed by ultrahigh-performance liquid chromatography (UPLC) (Waters, Japan) and Water Xeveo G2 time-of-flight mass spectrometer (qTOF-MS) (Waters, Japan). The chromatographic conditions were as following: A%: 0.05% formic acid–water; B%: 0.05% formic acid–acetonitrile; *m/z*: 120–1,200; positive scan mode, chromatographic columns: ACQUIT UPLC-BEH C18 Column (1.7 mm, 2.1 mm, × 50 mm). Leu-Enkephalin was applied as the internal standard.

### Statistical Analysis

GC-MS and LC-qTOF-MS results were imported into the SIMCA-P14 software package (Umetrics, Umeå, Sweden). Orthogonal partial least squares discriminant analysis (OPLS-DA) was performed to visualize differences between groups after mean centering and unit variance scaling. Significantly different metabolites were screened by the multivariate statistical method and Student’s *t*-test (VIP > 1.0 and *P-*value < 0.05). A 7-round crossvalidation was applied, with 1/7 of the samples being excluded from the mathematical model in each round to avoid overfitting. Significantly different metabolites were subjected to Kyoto Enrichment of Genes and Genomes (KEGG) enrichment. SPSS version 24.0 software (Chicago, IL, United States) was applied to calculate the score of principal component “Q.” A log2 transformation was conducted to improve data normality. Min–max normalization was additionally tested. Hierarchical clustering analysis was performed with R-3.2. Heatmaps, histograms, and pathway maps were drawn with R-3.2 language software, GraphPad Prism8, and Visor, respectively.

## Results

### The Saline–Alkali Degree of the Rhizosphere Community

**FIGURE 1:**
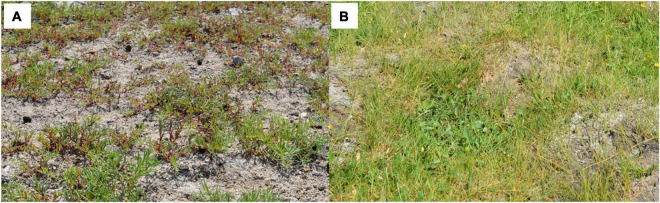
The habitats of *S. salsa* community and *P. tenuiflora* community: **(A)** *S. salsa* community; **(B)** *P. tenuiflora* community.

*Suaeda salsa* community (Figure 1A) and *P. tenuiflora* community (Figure 1B) are pioneer communities for saline–alkali land improvement in Hulun Buir Grassland. Measured indicators of the rhizosphere soils around *S. salsa* and *P. tenuiflora* community demonstrated that both communities showed high pH values and could be defined as salinization soils according to soil alkalization classification standard (SSC) (Table 1). The amounts of anions indicated that *S. salsa* community rhizosphere soil could be identified as the saline–alkali soil of chloride whereas *P. tenuiflora* community could be identified as saline–alkali soil of sulfate.

**TABLE 1:** The values of soil indicators of *S. salsa* community and *P. tenuiflora* community.

	**pH**	**ESP (%)**	**SSC (%)**	**EC (mS/cm)**	**SO4^2^**^–^ **(cmol/kg)**	**Cl**^–^ **(cmol/kg)**	**CO_3_^2^**^–^ **(cmol/kg)**	**HCO_3_**^–^ **(cmol/kg)**	**(CO_3_^2^**^–^ **HCO_3_^–^)/ (Cl^–^+SO_4_^2–^)**	**Cl^–^/SO4^2–^**
Ss	9.42	54.87	0.63	1,074	3.07	2.92	0.72	0.20	0.24	1.34
Ps	8.76	40.58	0.57	895	2.85	0.81	2.52	0.35	0.81	0.37

*Ss, the rhizosphere soil around S. salsa; Ps, the rhizosphere soil around P. tenuiflora; ESP, exchangeable sodium percentage; SSC, Soluble salt content; EC, electrical conductivity.*

Moreover, these recorded parameters of rhizosphere soils implied that *S. salsa* community is facing more severe alkalinity stress than *P. tenuiflora* community. It was consistent with the observations that the rhizosphere soil around *P. tenuiflora* seemed to be more fertile and less exposed than the rhizosphere soil around *S. salsa* (Figure 1). *S. salsa* community even presented “leucophylline” and harden into a lump.

### The Accumulation of Main Elements in *S. salsa* Community and *P. tenuiflora* Community

**FIGURE 2:**
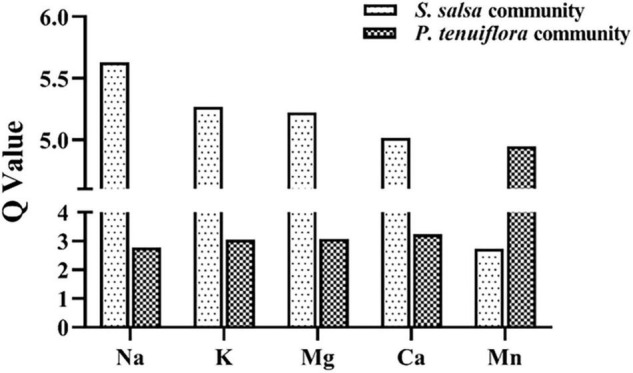
The *Q*-values of significantly different elements between *S. salsa* community and *P. tenuiflora* community.

To explore the influences of elements on plant communities on saline–alkali stress, we tested 11 kinds of main elements in plants and rhizosphere soils, including K, Ca, Na, Mg, Fe, Zn, Cu, Ni, Mn, Mo, and B. Among them K, Ca, Na, Mg, and Mn, were discovered to be significantly different between *S. salsa* community and *P. tenuiflora* community by the analysis of OPLS-DA (Supplementary Figure 1A and Supplementary Table 1). Calculation of *Q*-values of these significantly different elements showed that *Q*-values of K, Na, Mg, and Ca were obviously higher in *S. salsa* community as compared with in *P. tenuiflora* community (Figure 2). The only microelement that was significantly accumulated in *P. tenuiflora* compared with *S. salsa* community was Mn (Figure 2). These observations implied that *S. salsa* community has stronger capabilities of element adsorption, mobilization, and utilization under saline–alkali stress.

**FIGURE 3:**
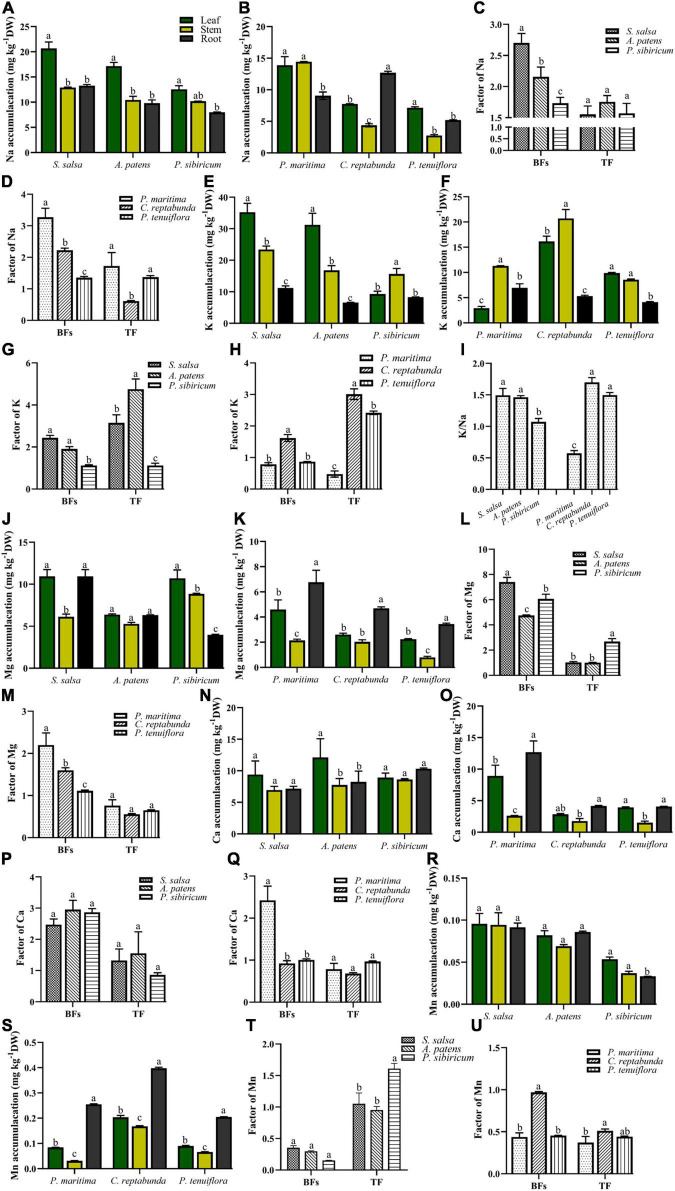
The accumulation of significantly different elements in various kinds of plants in *S. salsa* community and *P. tenuiflora* community: **(A,E,J,N,R)** Na, K, Mg, Ca, Mn accumulation in *S. salsa* community, respectively; **(B,F,K,O,S)** Na, K, Mg, Ca, Mn accumulation in *P. tenuiflora* community, respectively; **(C,G,L,P,T)** the BFs and TF of Na, K, Mg, Ca, Mn in *S. salsa* community, respectively; **(D,H,M,Q,U)** the BFs and TF of Na, K, Mg, Ca, Mn in *P. tenuiflora* community, respectively. **(I)** The ratio of Na and K; The accumulation of significantly different elements is summarized from three biological replicates and presented as the mean ± standard error. Different letters indicate significant differences among treatments (*p* < 0.05).

In addition, the contents of K, Ca, Na, Mg, and Mn in the organs of different kinds of plants in *S. salsa* community and *P. tenuiflora* community were measured. In *S. salsa community*, Na was mainly accumulated in the leaves of *S. salsa* and *P. sibiricum* (Figure 3A). In *P. tenuiflora* community, Na was enriched in the stems of *P. maritima* and the roots of *C. reptabunda* (Figure 3B). Calculated BFs showed that community plants obtained abundant Na from soils (BFs > 1) (Figures 3C,D). Plants in *S. salsa* community had relatively higher TF values as compared with plants in *P. tenuiflora* community, suggesting that plants in the *S. salsa* community had stronger Na transport capacities (Figures 3C,D).

Measurement of the contents of K demonstrated that K was mainly enriched in aboveground parts of plants of *S. salsa* community (Figure 3E). The amount of K was less in *P. tenuiflora* community and significantly accumulated in the stems of plants in *P. tenuiflora* community (Figure 3F). Therefore, the transport capacity of K in *P. tenuiflora* community might be lower than that in *S. salsa* community. BFs in all tested *S. salsa* community plants and *C. reptabunda* in *P. tenuiflora* community were higher than 1, indicating that these plants are K hyperaccumulator plants (Figures 3G,H). The high TF values of these plants suggested that *S. salsa*, *A. patens*, *C. reptabunda*, and *P. tenuiflora* have high K transport capacity from roots to aboveground parts of plants (Figures 3G,H). K/Na ratio, an important index of plants to saline–alkali stress, was further calculated. High K/Na ratios were observed in all plants expect for *P. maritime* in *P. tenuiflora* community, demonstrating that these plants ensure the absorption of K under saline–alkali stress (Figure 3I). Moreover, higher K/Na values in *S. salsa* community as compared with in *P. tenuiflora* community implied that the mobilization and transportation of K in *S. salsa* community are more active to saline–alkali stress whereas plants in *P. tenuiflora* community are more tolerant to saline–alkali stress.

Magnesium was found to be mainly concentrated in all organs of plants in *S. salsa* community (Figure 3J) and the roots of plants in *P. tenuiflora* community (Figure 3K). High BFs and low TF values of these plants revealed that *S. salsa* community and *P. tenuiflora* community plants can absorb a large number of Mg from soil and store Mg in roots (Figures 3L,M). Ca seemed to be equally distributed in leaves, stems, and roots of plants in *S. salsa* community (Figure 3N) as well as the leaves and roots in *P. maritima* in *P. tenuiflora* community (Figure 3O). BFs and TF values revealed that Ca mainly plays biological roles in leaves and roots in community plants (Figures 3P,Q).

Different from the low abundance of Mn in *S. salsa* community (Figure 3R), Mn was discovered to be accumulated in the plants in *P. tenuiflora* community, especially roots (Figure 3S). The patterns of BFs and TF values were also different in *S. salsa* community and *P. tenuiflora* community (Figures 3T,U). High levels of TF values in *S. salsa* community indicated that Mn is mainly transported to aboveground parts of plants in *S. salsa* community (Figure 3T). On the other hand, low levels of TF values in *P. tenuiflora* community suggested that the absorbance and transportation of Mn to aboveground organs is less robust in *P. tenuiflora* community (Figure 3U).

### The Response of Primary Metabolisms to Saline–Alkali Stress

**FIGURE 4:**
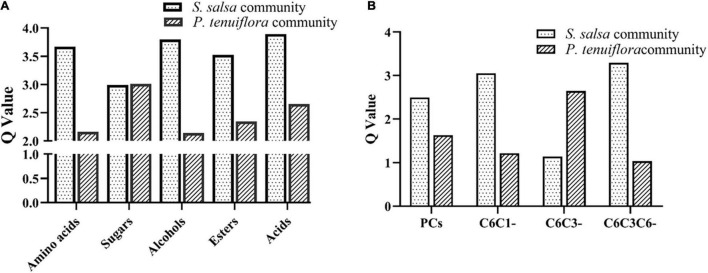
The *Q*-values of significantly different metabolisms between *S. salsa* community and *P. tenuiflora* community: **(A)** the *Q*-values of significantly primary different metabolisms between *S. salsa* community and *P. tenuiflora* community; **(B)** the *Q*-values of a phenolic compounds C6C1-compounds, C6C3-compounds, and C6C3C6-compounds; PCs, phenolic compounds.

GC-MS detection of primary metabolisms also revealed the differences between *S. salsa* community and *P. tenuiflora* community. Among 226 measured metabolisms, a total of 68 primary metabolites showed significantly different expression patterns between *S. salsa* community and *P. tenuiflora* community by analysis of OPLS-DA (Supplementary Figure 1B). These primary metabolisms were classified to seven sugars, seven amino acids, 12 alcohols, six esters, four amines, and 32 acids (including 10 phenolic acids and 22 non-phenolic compounds) (Supplementary Table 2). *Q*-values of primary metabolisms were calculated based on each class. *Q*-values of amino acids, alcohols, esters, and acids were significantly higher in *S. salsa* community (Figure 4A). *Q*-values of sugars were similar in *S. salsa* community and *P. tenuiflora* community (Figure 4A). Notably, soluble sugars such as tagatose, D-talose, and trehalose were more accumulated in *P. tenuiflora* community (Supplementary Table 2). It suggested that soluble sugars play an important role in *P. tenutiflora* community to respond to saline–alkali stress.

### The Response of Phenolic Compounds to Saline–Alkali Stress

Considering the importance of acids, especially phenolic compounds, under saline–alkali stress, phenolic compounds were investigated in detail. A total of 34 important phenolic compounds were detected. A total of 24 phenolic compounds were found to be accumulated in our community plants and a total of 11 significantly different metabolisms were screened by OPLS-DA (Supplementary Figure 1C). Differences of acids between *S. salsa* community and *P. tenuiflora* community were mainly due to differences in phenolic compounds (Figure 4B). These differentially expressed phenolic compounds could be classified to C6C1-compounds, C6C3-compounds, and C6C3C6-compounds. C6C1-compounds and C6C3C6-compounds were more accumulated in *S. salsa* community whereas C6C3-compounds were more accumulated in *P. tenuiflora* community (Figure 4B).

**FIGURE 5:**
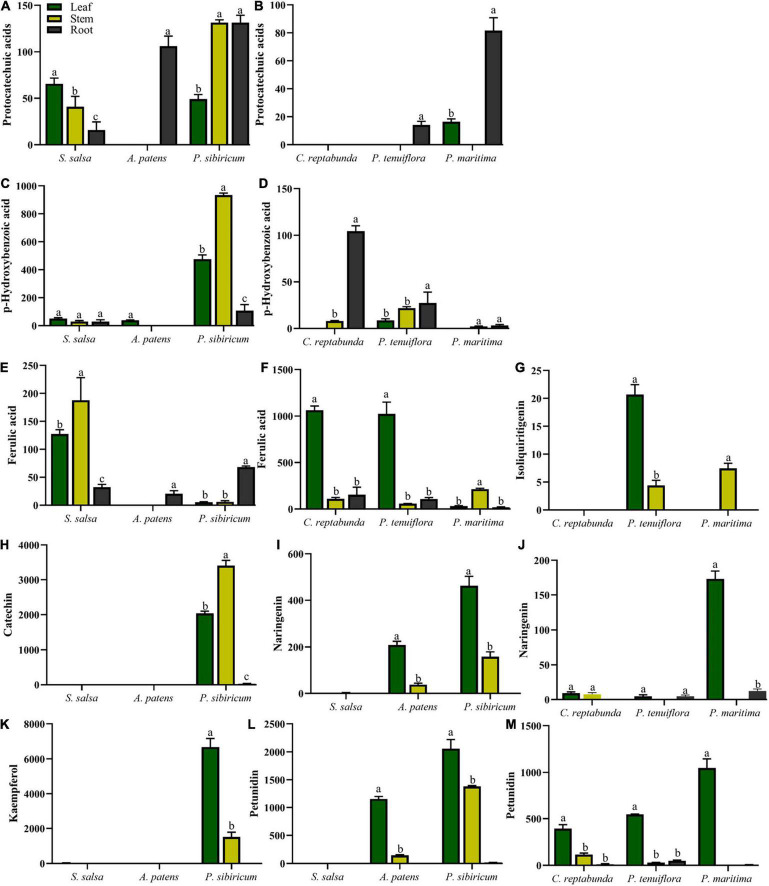
Relative contents of significantly different phenolic compounds in *S. salsa* community and *P. tenuiflora* community: **(A,C)** relative contents of C6C1-compounds in *S. salsa* community; **(B,D)** relative contents of C6C1-compounds in *P. tenuiflora* community; **(E)** relative contents of C6C3-compounds in *S. salsa* community; **(F,G)** relative contents of C6C3-compounds in *P. tenuiflora* community **(H,I,K,L)** relative contents of C6C3C6-compounds in *S. salsa* community **(J,M)** relative contents of C6C3C6-compounds in *P. tenuiflora* community; The accumulation of significantly different elements is summarized from 3 biological replicates and presented as the mean ± standard error. Different letters indicate significant differences among treatments (*p* < 0.05).

The distributions of C6C1-compounds in plant organs showed that C6C1-compound protocatechuic acid was mainly gathered in the stems and roots of *A. patens* and *P. sibiricum* in *S. salsa* community (Figure 5A). In *P. tenuiflora* community, protocatechuic acid was more accumulated in the roots of *P. maritima* (Figure 5B). Another C6C1-compound, p-hydroxybenzoic acid, was mainly accumulated in the aboveground of *P. sibiricum* in *S. salsa* community and also in the roots of *C. reptabunda* in *P. tenuiflora* community (Figures 5C,D). C6C3-compound ferulic acid was mainly concentrated in the aboveground part of our community plants (Figures 5E–G). It was significantly accumulated in *P. tenuiflora* community, especially the leaves of *C. reptabunda* and *P. tenuiflora* (Figure 5F). C6C3C6-compounds were evidently enriched in the aboveground part of *A. patens* and *P. sibiricum* in *S. salsa* community (Figures 5H–M). Catechin and kaempferol were only detected in *P. sibiricum* in *S. salsa* community. The amounts of naringenin and pelunidin were also higher in *P. sibiricum* in the *S. salsa* community.

## Discussion

**FIGURE 6:**
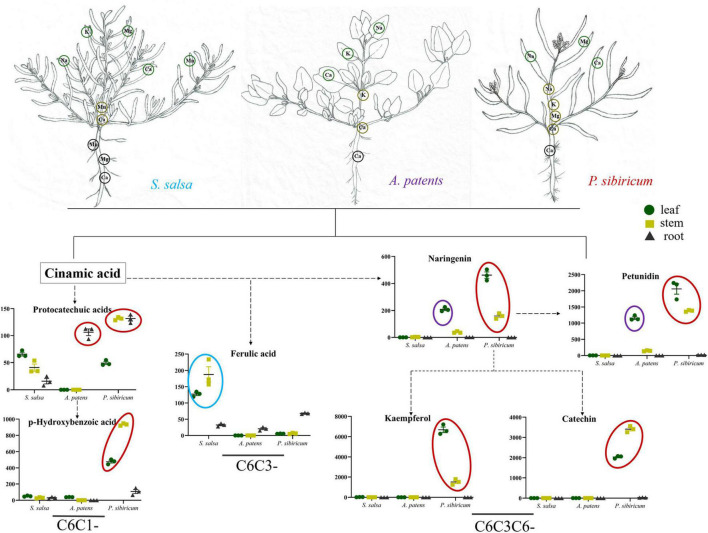
Visualization of pathway maps of significantly different elements and phenolic metabolites in *S. salsa* community. Blue circle, highly expressed in *S. salsa*, purple circle, highly expressed in *A. patents*, red circle, highly expressed in *P. sibiricum*. The green circle, yellow circle, and black circle represent leaf, stem and root, respectively.

**FIGURE 7:**
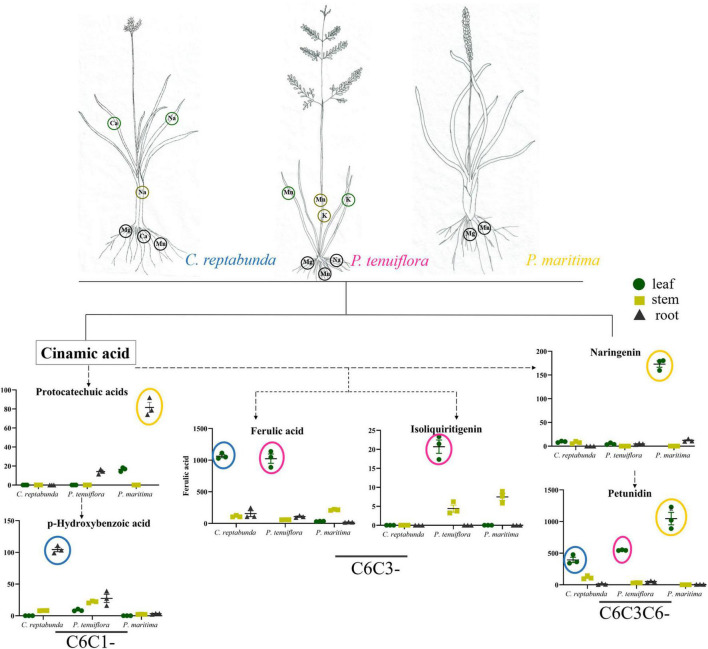
Visualization of pathway maps of significantly different elements and phenolic metabolites in *P. tenuiflora* community. Blue circle, highly expressed in *C. reptabunda*, pink circle, highly expressed in *P. tenuiflora*, orange circle, highly expressed in *P. maritime*. The green circle, yellow circle, and black circle represent leaf, stem and root, respectively.

Soil salinization is a major abiotic environmental factor that limits land utilization efficiency (Abdel and Tran, 2016). Given that Hulun Buir Grassland is influenced by obviously gradient salinization, here, we collected the rhizosphere soil of *S. salsa* community and *P. tenuiflora* community in Hulun Buir Grassland. We found that the rhizosphere soil of *S. salsa* community was chloride saline–alkali soil (Cl^–^ and SO_4_^2–^) and the rhizosphere soil of *P. tenuiflora* community was saline–alkali sulfate soil (SO_4_^2–^ and CO_3_^2–^). Moreover, the unique features of *S. salsa* community and *P. tenuiflora* community were characterized by determining the amounts of elements and primary metabolisms, especially phenolic compounds (Figures 6, 7).

### Element Response

Sodium is one of the most dominating toxic ions that induce soil salinization. Extremely accumulated Na can induce ionic imbalance and disrupt normal physiological metabolisms (Lin et al., 2018; He et al., 2020). Our observations showed that Na was significantly accumulated in plants in both *S. salsa* community and *P. tenuiflora* community, indicating that these plants can survive from excess Na from soils. Na was found to be transported to the aboveground parts of plants and accumulated in leaves and stems. High K^+^ and low Na^+^ concentrations are critical for maintaining osmotic pressure. The accumulation of Na in *S. salsa* community and *P. tenuiflora* community implied that the osmotic balance might be disturbed. Since there exists no special Na transportation channel, a competitive relationship between K (or other ions) and Na may be present during K/Na uptake under saline–alkali stress. Excessive Na^+^ can damage metabolic pathways by replacing K^+^ in key enzymatic reactions, causing the dysregulation of various biological processes (Guo et al., 2017). However, our results showed that K was also significantly accumulated in the aboveground parts of community plants, indicating that the accumulation of Na may not affect the absorption of K. The value of K/Na ratio suggested that community plants can grow normally under saline–alkali stress. Different from reducing Na uptake to alleviate injuries to plants, K uptake of community plants are seemingly not affected. Therefore, Na may be necessary for the growth of some halophytes.

Salt overly sensitive pathway is an important ionic stress signaling pathway to maintain Na/K balance by extruding Na^+^ into the apoplast. Ca^2+^ is a key signaling component in this pathway. Ca accumulation in tissues can instantly trigger the SOS–Na^+^ system to exclude and diminish the damage caused by Na toxicity (Jia et al., 2019). The significant accumulation of Ca in plant leaves and roots may activate the SOS–Na^+^ system. Moreover, accumulated Ca can enhance Ca^2+^ binding to Ca^2+^ sensor proteins and further support Na^+^ detoxification (Jia et al., 2019). Ca not only contributes to the SOS–Na^+^ signaling pathway, but also plays a pivotal role in cellular structure maintenance under saline–alkali stress. The accumulation of Ca helps to increase membrane stability under adverse conditions such as osmotic stress (Zeng et al., 2015). Ca accumulated in roots can function as a Ca storage and provide a Ca gradient for multiple biological processes (An et al., 2020). Our results implied that Ca may play an important role in plant saline–alkali tolerance by increasing the levels of Ca in roots, rather than by reducing the levels of Na.

It has been reported that saline–alkali stress can lead to decreased plant biomass and reduced photosynthesis (Guo et al., 2017; Nie et al., 2018). Mg participates in chlorophyll biosynthesis and affects photosynthetic rate. Superfluous Na and high pH value can result in deficiency of Mg, affecting the structure of chloroplast and the activation of chlorophyllase enzyme. Our results showed that Mg was strongly absorbed from soil by community plants. The entry of Mg^2+^ into root cells is passively mediated *via* Mg^2+^ permeable channels. Saline–alkali stress may activate Mg^2+^-uptaking channels in roots. Chloroplast membrane-localized Mg-chelatase H subunit has been demonstrated as a key putative ABA receptor with essential roles in ABA signaling. Plant roots can perceive and defend against saline–alkali stress *via* ABA signaling (Jia et al., 2019). The accumulation of Mg^2+^ in roots of *S. salsa* community plants may regulate ABA signaling transduction and ABA receptor synthesis.

Manganese is also a key element involved in photosynthesis. Here, we identified the enrichment of Mn in *P. tenuiflora* community. Mn in leaves protects PSII cluster on photosynthetic electron transport whereas Mn in roots improves the resistance to saline–alkali stress by functioning as a component of Mn-SOD (Jia et al., 2019; Zhu et al., 2020).

It is worth noting that our comparison of elements in *S. salsa* community and *P. tenuiflora* community demonstrated different element strategies between these community plants. In *S. salsa* community plants, macroelements play significant roles under stress. Specifically, K is transported to the aboveground part to maintain high K^+^ and low Na^+^ concentrations and support normal physiological activity. Ca is used for triggering the SOS–Na^+^ system to efflux Na and improve membrane stability. Mg, as a Mg-chelatase H subunit of ABA receptor, plays a role in perceiving and defending against the saline–alkali stress. In *P. tenuiflora* community, the high abundance of microelement Mn in roots revealed the potential involvement of Mn–SOD in improving the resistance to saline–alkali stress.

### Phenolic Compounds Response

Phenolic compounds play primary roles in the regulation of plant’s life cycle and growth as well as the acceptance of the environmental challenge. They are the most widely distributed metabolites that are involved in environment responses (Liu et al., 2016; Liu J. et al., 2020). Phenolic compounds are referred to as cyclic compounds containing at least one hydroxyl group attached to an aromatic ring and can be divided into three major groups, i.e., C6C1-compounds, C6C3-compounds, and C6C3C6-compounds, according to the number and binding position of convertible hydroxyl groups on the aromatic chain. C6C1-compounds, such as protocatechuic acid and hydroxybenzoic acid, have C6C1 carbon skeletons derived from cinnamic acid. C6C3-compounds, such as ferulic acid and p-hydroxycinnamic acid, have C6C3 carbon skeleton derived from phenylalanine. C6C3C6-compounds (also named flavonoids), such as naringenin and kaempferol, have C6C3C6 carbon skeletons (Liu et al., 2016; Wu et al., 2018; Liu Y. et al., 2020). Phenolic compounds are able to scavenge reactive oxygen species (ROS) and respond to stressful environmental conditions, such as drought, temperature extremes, and high ultraviolet radiation (Heleno et al., 2015). Some certain species of plants even develop phenolic compounds to inhibit the growth of other competitors (Heleno et al., 2015; Liu Y. et al., 2020).

Some previous studies have demonstrated that organic acid metabolisms can help many plants, such as *Chloris virgata*, *Suaeda salsa*, and *Leymus chinensis*, to adapt to alkali stress (Yang et al., 2008, 2020; Lin et al., 2014). Similar to their observations, our results showed that 32 acids were enriched in differentially expressed primary metabolisms in *S. salsa* community and *P. tenuiflora* community, accounting for half of the significantly expressed metabolisms. The high levels of organic acids may be key adaptive mechanisms that help to stabilize the intracellular pH, neutralize excess toxic cations, and maintain ionic balance (Guo et al., 2017). The secretion of organic acids may make the rhizosphere to be more acidic, helping the rhizosphere to neutralize surrounding alkalinity (Zhao et al., 2019). Our results demonstrated that phenolic compounds occupied a large proportion in differentially expressed acids between *S. salsa* community and *P. tenuiflora* community. C6C1-compounds and C6C3C6-compounds were more accumulated in *S. salsa* community. C6C1-compounds, usually induced by biotic elicitors, are commonly used as signaling molecules to defend stress. We speculate that in *P. sibiricum*, activated p-hydroxybenzoic acid functions as a signaling cascade and communicates with C6C3C6-compounds. There may also be communications between p-hydroxybenzoic acid and protocatechuic acid. Communication signaling was magnified in the aboveground part of plants *via* p-hydroxybenzoic acid, and affected C6C3C6-compounds accumulated in the aboveground in *P. sibiricum* (Figure 7). Therefore, there may exist a continuity from C6C1-compounds to C6C3C6-compounds. C6C3C6-compounds accumulated in the epidermal layers of leaves and stems act as filters and absorb radiation in the UV-B portion of the spectrum (Agati et al., 2011; Liu Y. et al., 2020). We speculate that C6C3C6-compounds block the toxicity of saline–alkali *via* a similar way. Excess Na^+^ or ROS can be chelated in the epidermal layers by C6C3C6-compounds to protect cells. Increased C6C3C6-compounds may be due to their strong defense and antioxidant capacity (Liu Y. et al., 2020). We speculate that the distributions of the carbon skeleton of phenolic compounds are “primed” by the mechanisms that involve metabolism reprogramming. It will help *S. salsa* community plants to cope with saline–alkali stress.

The relative contents of C6C1-compounds in *P. tenuiflora* community were far below their contents in *S. salsa* community, indicating that the responses of phenolic compounds in *P. tenuiflora* community are weaker than that in *S. salsa* community. Interestingly, the resources of the carbon skeleton flow more to the C6C3 pathway in *P. tenuiflora* community. The roles of C6C3-cmpounds are different from C6C3C6-compounds. C6C3-cmpounds may only have basic reducibility to eliminate free radicals and maintain cell morphology. Therefore, different response modes of phenolic compounds may be applied in *S. salsa* community and *P. tenuiflora* community. In *S. salsa* community, C6C1-compounds stimulate C6C3C6-compounds to block toxicity whereas in *P. tenuiflora* community, C6C1-compounds induce C6C3-compounds to tolerate stress.

Collectively, in our current study, by applying the community as a unit instead of focusing on a single plant, We achieved characteristics of plant communities, built a conceptual model of community plant cooperation under saline–alkali stress, and provided a novel way for the investigation of response strategies against saline–alkali stress.

## Data Availability Statement

The original contributions presented in the study are included in the article/Supplementary Material, further inquiries can be directed to the corresponding author/s.

## Author Contributions

QC and XL designed the research and wrote the manuscript with the contribution of YJ, ZZ, and MC performed part of the experiments. XG, JZ, and ZT performed part of the data analysis. All authors have read and approved the manuscript.

## Conflict of Interest

The authors declare that the research was conducted in the absence of any commercial or financial relationships that could be construed as a potential conflict of interest.

## Publisher’s Note

All claims expressed in this article are solely those of the authors and do not necessarily represent those of their affiliated organizations, or those of the publisher, the editors and the reviewers. Any product that may be evaluated in this article, or claim that may be made by its manufacturer, is not guaranteed or endorsed by the publisher.

## Supplementary Material

Supplementary Figure 1 The OPLS-DA score plot between *S. salsa* community and *P. tenuiflora* community. **(A)** The OPLS-DA score plot of element metabolisms, **(B)** The OPLS-DA score plot of primary metabolisms, **(C)** The OPLS-DA score plot of phenolic compounds, Green circle: *S. salsa* community, blue circle: *P. tenuiflora* community.

## References

[B1] Abdel L. Tran L. P. (2016). Impacts of priming with silicon on the growth and tolerance of maize plants to alkaline stress. *Front. Plant Sci.* 7:243. 10.3389/fpls.2016.00243 27014283 PMC4785188

[B2] Agati G. Cerovic Z. G. Pinelli P. Tattini M. (2011). Light-induced accumulation of ortho-dihydroxylated flavonoids as non-destructively monitored by chlorophyll fluorescence excitation techniques. *Environ. Exp. Bot.* 73 3–9. 10.1016/j.envexpbot.2010.10.002

[B3] An Y. Yang X. Zhang L. Zhang J. Guo C. (2020). Alfalfa MsCBL4 enhances calcium metabolism but not sodium transport in transgenic tobacco under salt and saline-alkali stress. *Plant Cell Rep.* 39 1–15. 10.1007/s00299-020-02543-x 32333150

[B4] Ban Z. Q. (2007). Microarray and suppression subtractive hybridization analyses of gene expression in *Puccinellia tenuiflora* after exposure to NaHCO3. *Plant Sci.* 173 309–320. 10.1016/j.plantsci.2007.06.011

[B5] Bielicka-Daszkiewicz K. Hadzicka M. Voelkel A. (2012). Optimization of SPE/GC/HPLC analytical procedure for determination of phenol, quinones, and carboxylic acids in water samples. *ISRN Chromatogr.* 14 1–7. 10.5402/2012/680929

[B6] Chen Q. Lu X. Y. Guo X. R. Li D. W. Guo Q. X. (2017). Metabolomics characterization of two apocynaceae plants, Catharanthus roseus and Vinca minor, using GC-MS and LC-MS methods in combination. *Molecules* 22 997–1013. 10.3390/molecules22060997 28629120 PMC6152753

[B7] Chen Q. Lu X. Y. Guo X. R. Pan Y. J. Yu B. F. Tang Z. H. (2018). Differential responses to Cd stress induced by exogenous application of Cu, Zn or Ca in the medicinal plant *Catharanthus roseus*. *Ecotoxicol. Environ. Saf.* 157 266–275. 10.1016/j.ecoenv.2018.03.055 29626640

[B8] Chen Q. Lu X. Guo X. Xu M. Tang Z. (2021). A source-sink model explains the difference in the metabolic mechanism of mechanical damage to young and senescing leaves in *Catharanthus roseus*. *BMC Plant Biol.* 21:154. 10.1186/s12870-021-02934-6 33771114 PMC7995597

[B9] Guo R. Shi L. X. Yan C. Zhong X. Gu F. X. Liu Q. (2017). Ionomic and metabolic responses to neutral salt or alkaline salt stresses in maize (*Zea mays* L.) seedlings. *BMC Plant Biol.* 17:41. 10.1186/s12870-017-0994-6 28187710 PMC5301417

[B10] He K. He G. Wang C. Zhang H. Hu R. (2020). Biochar amendment ameliorates soil properties and promotes *Miscanthus* growth in a coastal saline-alkali soil. *Appl. Soil Ecol.* 155 103674. 10.1016/j.apsoil.2020.103674

[B11] Heleno S. A. Martins A. Queiroz M. J. R. P. Ferreira I. (2015). Bioactivity of phenolic acids: metabolites versus parent compounds: a review. *Food Chem.* 173 501–513. 10.1016/j.foodchem.2014.10.057 25466052

[B12] Hseu Z. Y. Chen Z. S. Tsai C. C. Tsui C. C. Cheng S. F. Liu C. L. (2002). Digestion methods for total heavy metals in sediments and soils. *Water Air Soil Pollut.* 141 189–205. 10.1023/A:1021302405128

[B13] Jia X. Zhu Y. Zhang R. Zhu Z. Zhu Z. L. Zhao T. (2019). Ionomic and metabolomic analyses reveal the resistance response mechanism to saline-alkali stress in *Malus halliana* seedlings. *Plant Physiol. Biochem.* 147 77–901. 10.1016/j.plaphy.2019.12.001 31846851

[B14] Kobayashi S. Abe N. Yoshida K. T. Liu S. Takano T. (2012). Molecular cloning and characterization of plasma membrane- and vacuolar-type Na/H antiporters of an alkaline-salt-tolerant monocot, *Puccinellia tenuiflora*. *J. Plant Res.* 125 587–594. 10.1007/s10265-012-0475-9 22270695

[B15] Kore-Eda S. Cushman M. A. Akselrod I. Bufford D. Cushman J. (2004). Transcript profiling of salinity stress responses by large-scale expressed sequence tag analysis in *Mesembryanthemum crystallinum*. *Gene* 341 83–92. 10.1016/j.gene.2004.06.037 15474291

[B16] Lin J. X. Peng X. Y. Hua X. Y. Sun S. N. Wang Y. N. Yan X. F. (2018). Effects of arbuscular mycorrhizal fungi on *Leymus* chinensis seedlings under salt-alkali stress and nitrogen deposition conditions: from osmotic adjustment and ion balance. *RSC Adv.* 8 14500–14509. 10.1039/C8RA00721G PMC9079982 35540780

[B17] Lin J. Mu C. Wang Y. Li Z. Li X. J. (2014). Physiological adaptive mechanisms of leymus Chinensis during germination and early seedling stages under saline and alkaline conditions. *J. Anim. Plant Sci.* 24 904–912.

[B18] Liu J. Kang R. Liu Y. Wu K. X. Yan X. Song Y. (2020). Differential metabolite accumulation in different tissues of gleditsia sinensis under water stress and rehydration conditions. *Forests* 11 542–556. 10.3390/f11050542

[B19] Liu J. Liu Y. Wang Y. Zhang Z. H. Zu Y. G. Efferth T. (2016). The combined effects of ethylene and MeJA on metabolic profiling of phenolic compounds in *Catharanthus roseus* revealed by metabolomics analysis. *Front. Physiol.* 7:217. 10.3389/fphys.2016.00217 27375495 PMC4895121

[B20] Liu Y. Liu J. Abozeid A. Wu K. X. Guo X. R. Mu L. Q. (2020). UV-B radiation largely promoted the transformation of primary metabolites to phenols in *Astragalus mongholicus* seedlings. *Biomolecules* 10 504–525. 10.3390/biom10040504 32225015 PMC7226020

[B21] Lu X. Chen Q. Cui X. Abozeid A. Liu Y. (2021). Comparative metabolomics of two saline-alkali tolerant plants Suaeda glauca and *Puccinellia tenuiflora* based on GC-MS platform. *Formerly Nat. Prod. Lett.* 35 499–502. 10.1080/14786419.2019.1633647 31282217

[B22] Nie W. Gong B. Chen Y. Wang J. (2018). Photosynthetic capacity, ion homeostasis and reactive oxygen metabolism were involved in exogenous salicylic acid increasing cucumber seedlings tolerance to alkaline stress. *Sci. Hortic.* 235 413–423. 10.1016/j.scienta.2018.03.011

[B23] Pang Q. Zhang A. Zang W. Wei L. Yan X. F. (2016). Integrated proteomics and metabolomics for dissecting the mechanism of global responses to salt and alkali stress in *Suaeda corniculata*. *Plant Soil* 402 379–394. 10.1007/s11104-015-2774-0

[B24] Taji T. Seki M. Satou M. Sakurai T. Kobayashi M. Ishiyama K. (2004). Comparative genomics in salt tolerance between *Arabidopsis* and *Arabidopsis*-related halophyte salt cress using *Arabidopsis* microarray. *Plant Physiol.* 135 1697–1709. 10.1104/pp.104.039909 15247402 PMC519083

[B25] Wang W. D. Wang Y. H. Du Y. L. Zhao Z. Zhu X. J. Jiang X. (2014). Overexpression of *Camellia sinensis* H1 histone gene confers abiotic stress tolerance in transgenic tobacco. *Plant Cell Rep.* 33 1829–1841. 10.1007/s00299-014-1660-1 25063323

[B26] Wang Y. Chu Y. Liu G. Wang M. H. Jing J. Hou Y. (2007). Identification of expressed sequence tags in an alkali grass (*Puccinellia tenuiflora*) cDNA library. *J. Plant Physiol.* 164 78–89. 10.1016/j.jplph.2005.12.006 16545489

[B27] Wang Z. L. Li P. H. Fredricksen M. Gong Z. Z. Kim C. S. Zhang C. Q. (2004). Expressed sequence tags from *Thellungiella halophila*, a new model to study plant salt-tolerance. *Plant Sci.* 166 609–616. 10.1016/j.plantsci.2003.10.030

[B28] Wu K. X. Liu J. Liu Y. Guo X. R. Mu L. Q. Hu X. H. (2018). A comparative metabolomics analysis reveals the tissue-specific phenolic profiling in two acanthopanax species. *Molecules* 23 2078. 10.3390/molecules23082078 30127238 PMC6222473

[B29] Xia Z. Wei L. Wang Z. Wang T. J. (2013). Physiological and molecular features of *Puccinellia tenuiflora* tolerating salt and alkaline-salt stress. *J. Integr. Plant Biol.* 55 262–276. 10.1111/jipb.12013 23176661

[B30] Xu Z. Shao T. Lv Z. Yue Y. Liu A. Long X. (2020). The mechanisms of improving coastal saline soils by planting rice. *Sci. Total Environ.* 703:135529. 10.1016/j.scitotenv.2019.135529 31759722

[B31] Yang C. Shi D. Wang D. (2008). Comparative effects of salt and alkali stresses on growth, osmotic adjustment and ionic balance of an alkali-resistant halophyte *Suaeda glauca* (Bge.). *Plant Growth Regul.* 56 179–190. 10.1007/s10725-008-9299-y

[B32] Yang C. Zhao W. Wang Y. Zhang L. Huang S. Lin J. (2020). Metabolomics analysis reveals the alkali tolerance mechanism in *Puccinellia tenuiflora* plants inoculated with arbuscular mycorrhizal fungi. *Microorganisms* 8 327–347. 10.3390/microorganisms8030327 32110985 PMC7142761

[B33] Yang D. S. Zhang J. Li M. X. Shi L. X. (2017). Metabolomics analysis reveals the salt-tolerant mechanism in *Glycine soja*. *J. Plant Growth Regul.* 36 460–471. 10.1007/s00344-016-9654-6

[B34] Ye X. Wang H. Cao X. Jin X. Cui F. Q. Bu Y. (2019). Transcriptome profiling of Puccinellia tenuiflora during seed germination under a long-term saline-alkali stress. *BMC Genomics* 20:589. 10.1186/s12864-019-5860-5 31315555 PMC6637651

[B35] Yin Z. Zhang H. Zhao Q. Yoo M. J. Zhu N. Yu J. L. (2019). Physiological and comparative proteomic analyses of saline-alkali NaHCO3 -responses in leaves of halophyte *Puccinellia tenuiflora*. *Plant Soil* 437 137–158. 10.1007/s11104-019-03955-9

[B36] Yu J. J. Chen S. Wang T. Sun G. Dai S. J. (2013). Comparative proteomic analysis of *Puccinellia tenuiflora* leaves under Na2CO3 stress. *Int. J. Mol. Sci.* 14 1740–1762. 10.3390/ijms14011740 23322023 PMC3565345

[B37] Zeng Y. Li L. Yang R. Yi X. Zhang B. H. (2015). Contribution and distribution of inorganic ions and organic compounds to the osmotic adjustment in *Halostachys caspica* response to salt stress. *Sci. Rep.* 5:15867. 10.1038/srep13639 26350977 PMC4563356

[B38] Zhao Q. Suo J. Chen S. Jin Y. Ma X. Yin Z. P. (2016). Na2CO3-responsive mechanisms in halophyte *Puccinellia tenuiflora* roots revealed by physiological and proteomic analyses. *Sci. Rep.* 6:32717. 10.1038/srep32717 27596441 PMC5011731

[B39] Zhao Z. Liu J. Jia R. Bao S. Hai X. Chen X. J. (2019). Physiological and TMT-based proteomic analysis of oat early seedlings in response to alkali stress. *J. Proteomics* 193 10–26. 10.1016/j.jprot.2018.12.018 30576833

[B40] Zhu Y. Jia X. Wu Y. Hu Y. Cheng L. Zhao T. (2020). Quantitative proteomic analysis of Malus halliana exposed to salt-alkali mixed stress reveals alterations in energy metabolism and stress regulation. *Plant Growth Regul.* 90 205–222. 10.1007/s10725-019-00563-6

