# Supplementary figures and images for: Ionomic and Metabolomic Analyses Reveal Different Response Mechanisms to Saline–Alkali Stress Between Suaeda salsa Community and Puccinellia tenuiflora Community
Source: Front Plant Sci. 2021 Nov 30;12:774284. doi: 10.3389/fpls.2021.774284 (PMC8670416; doi:10.3389/fpls.2021.774284)

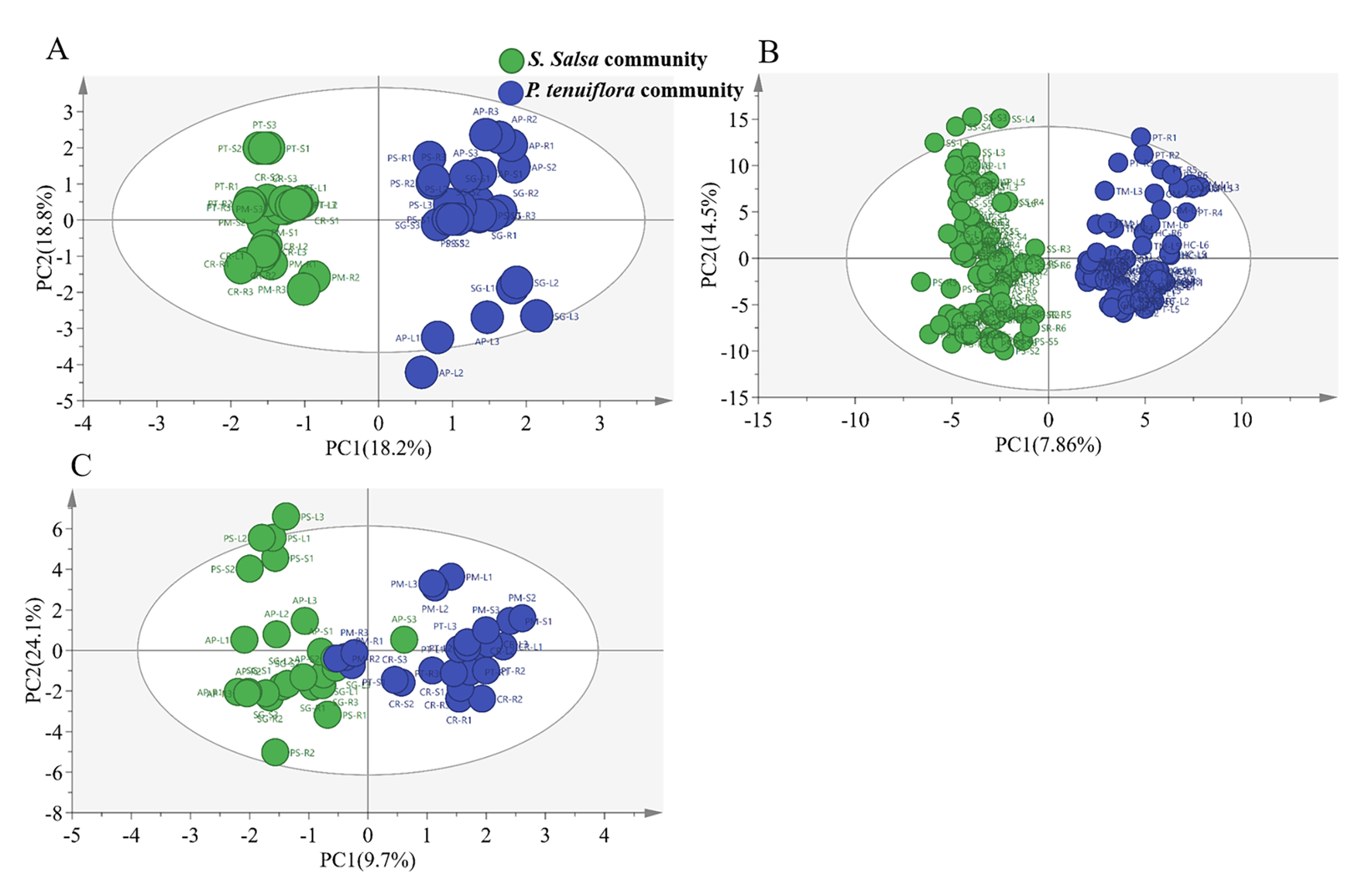

Supplement: Supplementary Figure 1 — The OPLS-DA score plot between S. salsa community and P. tenuiflora community. (A) The OPLS-DA score plot of element metabolisms, (B) The OPLS-DA score plot of primary metabolisms, (C) The OPLS-DA score plot of phenolic compounds, Green circle: S. salsa community, blue circle: P. tenuiflora community. [file Image_1.tif]
