# Supplementary material for: Ionomic and Metabolomic Analyses Reveal Different Response Mechanisms to Saline–Alkali Stress Between Suaeda salsa Community and Puccinellia tenuiflora Community
Source: Front Plant Sci. 2021 Nov 30;12:774284. doi: 10.3389/fpls.2021.774284 (PMC8670416; doi:10.3389/fpls.2021.774284)
Supplement: Supplementary file 2 [file Table_1.docx]

TABLE S1. List of significantly different elements between *S. salsa* community and *P. tenuiflora* community.

| Element | VIP | *P-value* |
| --- | --- | --- |
| Mg | 1.58 | ** |
| Ca | 1.35 | ** |
| Na | 1.24 | * |
| Mn | 1.11 | ** |
| K | 1.07 | * |

VIP, variable importance in the projection; *, *P-value* < 0.05; **, *P-value* < 0.01.
