# Supplementary material for: Ionomic and Metabolomic Analyses Reveal Different Response Mechanisms to Saline–Alkali Stress Between Suaeda salsa Community and Puccinellia tenuiflora Community
Source: Front Plant Sci. 2021 Nov 30;12:774284. doi: 10.3389/fpls.2021.774284 (PMC8670416; doi:10.3389/fpls.2021.774284)
Supplement: Supplementary file 3 [file Table_2.docx]

TABLE S2. List of significantly different metabolites between *S. salsa* community and *P. tenuiflora* community.

|  | | | | Metabolite | | VIP | *P*-value | Accumulated |
| --- | --- | --- | --- | --- | --- | --- | --- | --- |
| Amino acids (7) | | | | Oxoproline | | 1.01 | * | SG>PT |
|  | | | | N-Methyl-DL-alanine | | 1.54 | * | SG>PT |
|  | | | | Ornithine | | 1.40 | * | SG>PT |
|  | | | | 3-Hydroxynorvaline | | 1.36 | ** | SG>PT |
|  | | | | Nicotinoylglycine | | 1.24 | * | SG>PT |
|  | | | | N-Ethylglycine | | 1.20 | ** | SG>PT |
|  | | | | Pelargonic acid | | 1.02 | * | SG>PT |
| Sugars (7) | | | | Tagatose | | 1.78 | ** | SG<PT |
|  | | | | D-Talose | | 1.58 | * | SG<PT |
|  | | | | Trehalose | | 1.30 | ** | SG<PT |
|  | | | | Ribose | | 1.36 | * | SG>PT |
|  | | | | Gluconic lactone | | 1.23 | * | SG>PT |
|  | | | | Lyxose | | 1.07 | ** | SG>PT |
|  | | | | Fucose | | 1.04 | ** | SG>PT |
| Alcohols (12) | | | | Phytol | | 1.64 | ** | SG>PT |
|  | | | | 1-Hexadecanol | | 1.60 | * | SG>PT |
|  | | | | 2-Deoxyerythritol | | 1.40 | * | SG>PT |
|  | | | | 2-Aminoethanethiol | | 1.32 | ** | SG>PT |
|  | | | | 2-Deoxyerythritol | | 1.40 | * | SG>PT |
|  | | | | Benzyl alcohol | | 1.27 | * | SG>PT |
|  | | | | 1,2,4-Benzenetriol | | 1.14 | * | SG>PT |
|  | | | | Dithioerythritol | | 1.13 | * | SG>PT |
|  | | | | 1,5-Anhydroglucitol | | 1.12 | * | SG>PT |
|  | | | | 2-Amino-1-phenylethanol | | 1.00 | * | SG>PT |
|  | | | | Piceatannol | | 1.22 | ** | SG<PT |
|  | | | | Cuminic alcohol | | 1.20 | * | SG<PT |
| Esters (6) | | | | Methyl Phosphate | | 1.39 | * | SG>PT |
|  | | | | Methyl Octanoate | | 1.26 | * | SG>PT |
|  | | | | Nonanoic acid Methyl ester | | 1.23 | ** | SG>PT |
|  | | | | Methyl Heptadecanoate | | 1.16 | * | SG>PT |
|  | | | | Linoleic acid Methyl ester | | 1.12 | * | SG>PT |
|  | | | | D-Erythronolactone | | 1.05 | * | SG<PT |
| Amines (4) | | | | 5-Methoxytryptamine | | 1.60 | * | SG>PT |
|  | | | | Putrescine | | 1.01 | * | SG>PT |
|  | | | | N-2-Fluorenylacetamide | | 1.54 | * | SG>PT |
|  | | | | Asparagine | | 1.33 | * | SG<PT |
| Acids (non-phenolic compounds, 22) | | | | Glycolic acid | | 1.76 | * | SG>PT |
|  |  |  |  | Dehydroascorbic acid | | 1.70 | * | SG>PT |
|  |  |  |  | 3-Aminoisobutyric acid | | 1.65 | * | SG>PT |
|  | | | | Palmitic acid | | 1.42 | * | SG>PT |
|  | | | | Lauric acid | | 1.39 | * | SG>PT |
|  | | | | Creatine | | 1.59 | * | SG>PT |
|  | | | | Citraconic acid | | 1.67 | * | SG>PT |
|  | | | | 2-Methylfumarate | | 1.59 | * | SG>PT |
|  | | | | Stearic acid | | 1.42 | * | SG>PT |
|  | | | | Ribulose-5-phosphate | | 1.43 | * | SG>PT |
|  | | 5-Hydroxyindole-2-carboxylic acid | | | 1.03 | | * | SG>PT |
|  | 2-Amino-2-norbornanecarboxylic acid | | | | | 1.37 | * | SG>PT |
|  | | | | Oxalic acid | | 1.29 | * | SG>PT |
|  | | | | 2-Ketobutyric acid | | 1.23 | * | SG>PT |
|  | | | | Glucoheptonic acid | | 1.14 | * | SG>PT |
|  | | | | Phenylacetic acid | | 1.13 | ** | SG>PT |
|  | | | | O-Succinylhomoserine | | 1.56 | * | SG>PT |
|  | | | | Fumaric acid | | 1.18 | ** | SG>PT |
|  | | | | Gentisic acid | | 1.08 | * | SG<PT |
|  | | | | 2-Keto-L-gulonic acid | | 1.49 | * | SG<PT |
|  | | | | 3-Phenyllactic acid | | 1.44 | * | SG<PT |
|  | | | | 2-Furoic Acid | | 1.06 | ** | SG<PT |
| Acids  (Phenolic compounds, 10) | | | | Catechol | | 1.61 | * | SG>PT |
|  |  |  |  | 3,4-Dihydroxybenzoic acid | | 1.41 | * | SG>PT |
|  |  |  |  | 3-Hydroxybenzoic acid | | 1.28 | * | SG>PT |
|  | | | | Naringenin | | 1.07 | ** | SG>PT |
|  | | | 4-Hydroxy-3-methoxybenzoic acid | | | 1.02 | * | SG>PT |
|  | | | | Guaiacol | | 1.01 | * | SG>PT |
|  | | | | Benzoic acid | | 1.39 | * | SG>PT |
|  | | | | Hydrocinnamic acid | | 1.03 | * | SG<PT |
|  | | | | Epigallocatechin | | 1.04 | ** | SG<PT |
|  | | | | 4-Hydroxycinnamic acid | | 1.06 | * | SG<PT |

VIP, variable importance in the projection; *, *P-value* < 0.05; **, *P-value* < 0.01; SG: *S. salsa* community; PT: *P. tenuiflora* community.
